# Pesticide Exposure and Parkinson's Disease: A Qualitative Study of Experiences in Ireland

**DOI:** 10.1111/hex.70329

**Published:** 2025-10-14

**Authors:** Lucy M. Collins, Sarah Moore, Emma O'Shea, Aidan White, Aideen M. Sullivan

**Affiliations:** ^1^ Department of Anatomy and Neuroscience, School of Medicine University College Cork Cork Ireland; ^2^ Parkinson's Disease Research Cluster University College Cork Cork Ireland; ^3^ Department of Pharmacology and Therapeutics, School of Medicine University College Cork Cork Ireland; ^4^ Centre for Gerontology and Rehabilitation, School of Medicine University College Cork Cork Ireland

**Keywords:** Parkinson's disease, Personal Protective Equipment, pesticides

## Abstract

**Introduction:**

Parkinson's disease (PD) is a common neurodegenerative disorder, affecting an estimated 10 million people globally, and its incidence rate is rising rapidly. PD most commonly develops sporadically, with only 5%–10% of cases linked to genetic causes. Environmental factors, including exposure to pesticides, have been associated with a higher risk of PD. Multiple studies have shown an increased incidence of PD in geographical regions which have high levels of pesticide use. However, there is little data on links between pesticide exposure and PD in Ireland, and the experiences of Irish people with PD in relation to pesticides remain underexplored.

**Aim:**

To conduct a qualitative study to explore the experiences and perceptions of individuals in Ireland regarding the potential links between occupational and environmental pesticide exposure and PD.

**Methods:**

This qualitative study investigated the experiences of individuals with PD and their families in Ireland, regarding pesticide exposure. Semi‐structured interviews were conducted with 21 participants, including people with Parkinson's (PwP) and their close relatives. Purposive sampling was used to recruit participants from farming and rural communities. The semi‐structured interviews lasted 10–15 min, and data were transcribed and analysed using thematic analysis. The research adhered to ethical guidelines, including informed consent and confidentiality.

**Results:**

Three main themes were identiifed from the analysis: ‘Occupation‐Related Pesticide Exposure and PD Risk’, ‘Dangers of Pesticides, and Barriers to Personal Protective Equipment (PPE) Usage’, and ‘Family and Community Influence/Mixed Messaging about Pesticide Safety’. Participants frequently reported significant pesticide exposure due to occupational activities or living in agricultural areas. Many people highlighted inadequate use of PPE and described how exposure was often unregulated or unavoidable. Family and community contexts further compounded exposure risks.

**Conclusion:**

This study highlights the need for heightened awareness and education regarding risks associated with pesticide exposure. It reveals gaps in the application of protective measures and highlights a need for improved regulation of safety protocols.

**Patient or Public Contribution:**

The design and conduct of this study were supported by people with Parkinson's disease and their family members, as well as members of the public who have experience with pesticide use. In advance of the study, members of the Cork branch of Parkinson's Ireland reviewed the research question, methodology and interview schedules, and provided feedback on these, which was incorporated into the study.

**Abbreviations**
PD: Parkinson's disease
PwP: People with Parkinson's
PPE: Personal Protective Equipment
MPTP: 1‐Methyl‐4‐phenyl‐1,2,3,6‐tetrahydropyridine
MPP^+^
: Methyl‐4‐phenylpyridinium

## 1 Introduction

Parkinson's disease (PD) is the second most common neurodegenerative disease globally, with genetic and environmental risk factors that can lead to its onset [1]. It is characterised by the degeneration of dopamine neurons which project from the substantia nigra of the midbrain to the basal ganglia in the forebrain, a brain area responsible for control of motor function [2]. Loss of dopamine in this pathway results in the characteristic motor symptoms of PD. A connection between environmental causes and the neuropathology of PD was first identified in 1982, when drug abusers of a new synthetic heroin compound developed severe PD‐like symptoms [3]. Subsequent analysis revealed that the synthetic compound contained 1‐methyl‐4‐phenyl‐1,2,3,6‐tetrahydropyridine (MPTP) and that a metabolite of MPTP, called 1‐methyl‐4‐phenylpyridinium (MPP^+^), was responsible for the Parkinsonian effects experienced by these patients. MPP^+^ is a neurotoxin that selectively targets dopaminergic neurons, mimicking the neuropathology of PD. This observation meant that MPTP could therefore be used to induce PD‐like pathology and symptoms in laboratory models of PD, a practice that continues to this day [4, 5, 6].

The link between pesticide exposure and the risk of developing PD is of increasing interest, with more research showing that several widely‐used pesticides have specific toxic effects on dopamine neurons [4]. Rotenone is a naturally occurring isoflavone, which, although banned in some countries, is used as an insecticide in others, and has been shown to induce Parkinsonism in laboratory animals [7]. Low‐level exposure to rotenone causes aggregation of α‐synuclein in the rodent brain; these aggregates result in the death of nigrostriatal dopaminergic neurons, leading to PD‐like motor symptoms [8]. Therefore, administration of either rotenone or MPTP is commonly used to produce in vivo laboratory rodent models of PD [9]. In addition to these established links between pesticides and PD pathophysiology shown in laboratory settings, further evidence for a link between the risk of developing PD and exposure to certain pesticides, including rotenone and paraquat, has been obtained through both epidemiological and experimental research [5]. Worldwide application of pesticides has increased by 20% in the last decade [10]. Considering the increased use of pesticides in our ever‐growing human population, the routes of entry of these pesticides, their mechanisms of action in the brain and their ability to pass the blood–brain barrier, are being increasingly researched [4, 11]. The prevalence of PD is growing due to an ageing population, better detection methods, increased recognition of atypical, age‐related presentations of PD, and the increased use of and unregulated exposure to pesticides [12, 13]. Additionally, this rising incidence of PD poses a significant burden in terms of resources, cost and strain on caregivers [14, 15]. Therefore, understanding how pesticides contribute to the environmental factors of PD aetiology is essential for decreasing the risk of PD onset.

Studies in California, France and Brazil have shown a link between pesticide use in an area and the increased prevalence and faster progression of PD in the population of that area and of surrounding areas [16, 17, 18, 19]. These studies have also shown novel findings: co‐exposure to multiple pesticides presents a greater risk of developing PD compared to exposure to a single pesticide [19]. Such research has also highlighted that frequency of pesticide use and proximity to pesticides increase the age of PD onset, and emphasised the need to study gene–pesticide interactions to identify susceptible genomic profiles in certain individuals [20, 21].

However, no study has yet documented this link in Ireland, a country with a historical and continuing reliance on farming as its largest export category [22]. Considering that certain farming types could increase the incidence of PD, it is crucial to identify, and act on, this risk in Ireland [16]. Recognising this link, educating the relevant population and ensuring robust regulation of pesticide use in Irish farming is essential. To achieve this, it is vital to understand the current level of awareness, knowledge and use of protective measures among Irish farmers regarding the link between pesticide exposure and PD.

Although treatments are available for PD, current drugs have limitations, and there is currently no cure for this debilitating disorder. Limiting exposure of individuals to its known risk factors is crucial for reducing the global incidence of PD [23]. This thematic analysis of interviews with individuals who have experienced contact with pesticides was conducted with people recruited from within farming communities in Ireland. Our study aimed to provide insights into the experiences and perspectives of this cohort, through interviews addressing potential links between PD and pesticide use, a known risk factor. It explored the experiences and perceptions of individuals in Ireland regarding the potential link between occupational and environmental pesticide exposure and PD. Our study focused on individuals diagnosed with PD, agricultural workers and healthcare professionals, to assess exposure history, risk perceptions and potential policy gaps. The study aimed to contribute to public health awareness and to inform regulatory discussions on pesticide use.

## 2 Methods

### 2.1 Data Collection and Recruitment

Semi‐structured qualitative interviews were conducted, either in person or remotely, with 21 individuals with PD and those who had a close relative with PD. This was part of a larger research study on the incidence and awareness of PD and pesticide use, which included analysis of survey data from a large cohort (*n* = 707) of individuals within farming communities in Ireland [24]. This study was part of a mixed‐methods design, wherein the interview questions within the qualitative thematic analysis complemented the quantitative aspects of the larger study. The qualitative component served an exploratory function within the study. The conceptual framework used followed Braun and Clarke's reflexive thematic analysis [25], to identify, analyse and interpret patterns of meaning (themes) within data.

The initial design of this study was conducted in collaboration with the Cork branch of Parkinson's Ireland. People with Parkinson's (PwP) were involved in the co‐design of the aims and methodology of the study, as well as contributing to the overall research question. The aim of collecting this interview data was to gather and interpret the experiences of individuals with a PD diagnosis who were exposed to pesticides in their work or living environment, and to inform ongoing research and public awareness about the potential link between PD and pesticide use.

Participants were recruited either onsite at the Irish National Ploughing Championships (2022), via social media, or through newsletters disseminated by Parkinson's Ireland, the Irish Farmers' Association, or Teagasc. Purposive/convenience sampling methods were used to select participants, based on recruitment to a larger study by the authors at these events. Inclusion criteria were individuals aged over 18 years, with the ability to speak English fluently, who were living with or supporting a person living with PD. Individuals were recruited at the National Ploughing Championships, and follow‐up interviews were conducted in person or over a telephone call.

The interview questions were semi‐structured, allowing for flexibility in discussing participants' experiences with pesticide exposure, as PwP or as close relatives of those affected by PD. Interviews were conducted by two experienced female researchers, that is, Aideen Sullivan (PhD; Professor) and Lucy Collins Stack (PhD; postdoctoral researcher) via telephone and recorded for accuracy. The researchers did not have prior relationships with any interviewees before their participation. The purpose of the study was clearly explained, and participants clarified their understanding of this to the researchers. Informed consent was obtained from all participants before the commencement of the interview, and all data were anonymised. The duration of the interviews ranged from 10 to 15 min. Recruitment was stopped once data saturation was reached, that is, when researchers recognised that no new information was arising from subsequent interviews.

### 2.2 Ethical Considerations

Ethical approval for this study was obtained from the University College Cork Social Research Ethics Committee (Log no. 2021‐116). Informed consent was obtained from all individuals, ensuring that each participant understood the purpose of the study, how their data would be used, and their right to withdraw at any time. Confidentiality and anonymity were maintained, and data were securely stored to protect the participants' identities. All interviews were conducted with trust and respect between researchers and participants.

### 2.3 Data Analysis

Speech data from the interviews were transcribed into text using a dictation function in Microsoft 365 software. The anonymised transcriptions were stored securely in a restricted‐access folder on University College Cork's secure server. All personal data were redacted to ensure confidentiality.

Data were analysed using Braun and Clarke's approach to thematic analysis [25]. The initial step involved thoroughly reading and re‐reading the interview transcripts to gain a deep familiarity with the data. During this phase, preliminary notes and initial ideas were recorded. The interviews were then systematically coded by one individual, using an inductive, bottom‐up approach. This involved identifying and labelling significant segments of data relevant to the research questions.

Coding was conducted iteratively. Initial codes guided the analysis of subsequent interviews, with codes being refined and updated as new data emerged. This refinement process included consolidating similar codes, renaming codes for clarity and introducing new codes as needed. The process continued until a comprehensive coding framework was established.

Following the coding phase, the data were reviewed to identify patterns and themes, both within and across interviews. Codes were organised into categories, which were then synthesised into overarching themes through several rounds of review and discussion. This interpretative analysis was conducted collaboratively, with team discussions helping to identify the patterns in the data and their relevance to the research question.

## 3 Results

Data collected from a total of 21 individuals were included in this thematic analysis. Not all participants provided demographic profiles, as specific questions on demographics were not required.

According to our participants, the potential link between PD and pesticide exposure was identified in the following three themes. These themes were identified from the analysis of the data, reflecting the experiences of PwP in relation to pesticides: ‘Occupation‐Related Pesticide Exposure and PD Risk’, ‘ Dangers of Pesticides, and Barriers to PPE Usage’, and ‘Family and Community Influence/Mixed Messaging about Pesticide Safety’.

Two sub‐themes were identified for the first theme of Occupation‐Related Pesticide Exposure: ‘Type of Pesticide’ and ‘Frequency of Exposure’.

All themes and sub‐themes are outlined in Table 1.

**Table 1:** Themes and sub‐themes.

Theme	Sub‐theme	Testimonial #	#	Quote
Occupation‐Related Pesticide Exposure and PD Risk	Type of Pesticide Used	2, 3, 6, 7, 9, 11, 12, 21	2	‘agri chemicals and pesticides’
			3	‘glyphosate, insecticide and fungicides’
			6	‘Organophosphates (foxim, diazinon, tetrachlorvinphos), Organochlorines (DDT), Amitraz, Piretroids, Enilconazole)’.
			7	‘Dichlorodiphenyltrichloroethane [DDT] Copper sulphate, Glyphosate and many more herbicides, pesticides and a whole range of veterinary pharmaceuticals’.
			9	‘My father has been diagnosed with Parkinson's and was a sheep and tillage farmer for most of his life.’
			11	‘Reglone and Gramoxone’
			12	‘whatever is in Forefront, whatever is in it we don't know’
			21	‘A few times we did use “bluestone” sometimes. We mixed powders, I think they were blue and white powders’
	Duration and Frequency of Exposure	2, 4, 5, 6, 7, 9, 10, 14, 15, 17	2	‘I was a cereal grower all my working life and would have been a user of agri chemicals and pesticides for all those years.’
			4	‘A farmer had a lot of spraying in growing strawberries’
			5	‘During my 47 years of farming I have used different pesticide’
			6	‘during 15 years of my practice in Spain, until 2003, I was exposed 2‐3 times per week to insecticides and fungicides’
			7	‘I worked in the discipline of Botany and Plant Science … and have used and been in contact with quite a few chemicals used in weed and pest control for more than 50 years.’
			9	‘My father has been diagnosed with Parkinson's and was a sheep and tillage farmer for most of his life.’
			10	‘My husband is a 37 year old farmer that has been diagnosed with Parkinson's Disease this year’
			14	‘she is 73 now so she was diagnosed maybe about 13 years ago and she lives in a small cull de sac in (town name redacted), and 2 other people were diagnosed with Parkinson's three of them often wondered was it anything to do with the local fertilizer factory in (town name redacted), which is since closed but would have released a lot of chemicals back in the day’
			15	‘‐My mother was diagnosed with Parkinson's in her 70's… *‐Did they live or work on a farm?* *‐*Yes, they did *‐What type of farm did they work on?* ‐Dairy and sheep’
			17	‘It was my father's farm before me and I took over when he got older…. I was diagnosed with PD in 2013, it was early onset idiopathic PD. There is no history of PD in my family, not to my knowledge.’
Dangers of Pesticides and Barriers to PPE Usage		5, 6, 10, 11, 12, 17, 18, 19, 20, 21	5	‘During my 47 years of farming I have used different pesticides, often without mask or gloves, using a pump action garden sprayer. Knapsack was too heavy for me as I'm not tall.’
			6	‘I was exposed 2‐3 times per week to insecticides and fungicides (treatments to animals in form of baths…. At the time I was only using medical gloves, though I frequently ended up bathed myself ‐once you are surrounded by sheep shaking themselves, you get the bath too.’
			10	‘He did have a toxic type exposure to a pesticide last year‐ he was spraying without PPE and was very dizzy and weak afterwards, his chronic symptoms seemed to start after that exposure (dizziness, rigidity and tremor). We said this to our Consultant but he said there is no link.’
			11	‘About 1980, my Dad gave me a puffer canister of “ABOL derris dust” to control woodlice and ants. It was inclined to raise a dust cloud when used and I remember being concerned after inadvertently breathing in some of the dust. However, Dad assured me that I had no need to worry as it was a natural product which was safe to use. His view was not surprising because ABOL had been traditionally marketed as a “non‐poisonous insecticide”. Being confident of its safety, I continued to use it occasionally for about 20 years.’
			12	‘*Do people use the proper protective equipment when using these sprays?* ‐I think there is an increase in people using it yes. I have a spray cabinet at home, with all of the PPE in it. People are more aware now than they were 10 years ago, of the dangers’
			17	‘A cloth (like a head scarf) or a scarf was used when my father was spraying. When I took over later, I took a bit more precautions, like a proper mask and gloves.’
				‘On the farm, lime was used commonly as a disinfectant. It was scattered in cattle houses after they had been cleaned. You would throw it around and the only thing to protect you from the dust was your collar pulled up over your nose and mouth.’
				‘He studied agricultural sciences in college and is much more aware of the risks associated with farming. He sprays at home and wears a filtered mask and uses gloves. He wears “oilskins” that he washes after’
			18	‘Back in the day (1960's/70's) people didn't take much notice of “health and safety”. I can remember farmers mixing chemicals in tin baths with their hands, no gloves. When they would pull their hands out, they would be brown up to where it went in.’
			19	‘Since I took over, I have been very careful with sprays, and chemicals in general. I have an old tractor that I only use for spraying. I built a cab for it that is sealed, and the sprayer itself is outside on the back. Even with this I still wear glover and a mask if I am spraying.’
			20	‘Back then there was no real PPE like there is now, it was a scarf or a tea towel wrapped around your head covering your nose and mouth.’
			21	‘Me and my husband didn't really understand the dangers of these chemicals, but we were careful. When my son took over in the 1980's/90's, he was very careful with chemicals and sprays.’
Family and Community Influence/Mixed Messaging About Pesticide Safety		1, 3, 7, 8, 11, 14, 17, 18	1	‘I also live in the countryside where pesticides would be used’
			3	‘I live in a village surrounded by agricultural fields…. I smell pesticides in the air when they apply it—glyphosate, insecticide and fungicides—and several times I've been out cycling and have encountered a sprayer at close quarters.’
			7	‘I am also from a farming background and have used and been in contact with quite a few chemicals used in weed and pest control for more than 50 years.’
			8	‘I am a farmer's son and my late father had Parkinson's also. The farm I grew up on had a tillage operation that relied heavily on pesticides at the time.’
			11	‘My experience with toxic pesticides, fungicides and herbicides goes back to my early youth…. He was involved in numerous trials of Gramoxone, some of which are documented in the Irish Journal of Agricultural Research. Dad always had a company car for his work, which doubled as the family car.… He would often offload these and store them in the garage, sometimes for prolonged periods. I do not recall being involved with the use of Gramoxone, though I do recall containers of Reglone and Gramoxone being stored on the upper shelves in the garage…. Dad was very proud of his garden, and would regularly treat the roses against aphids and fungi and treat the paths and lawn against weeds.’
			14	‘she lives in a small cul de sac in (town name redacted), and 2 other people were diagnosed with Parkinson's three of them often wondered was it anything to do with the local fertilizer factory in (town name redacted), which is since closed but would have released a lot of chemicals back in the day’
			17	‘I was born on a farm, in the early 60's, and have lived there for all my life.’
			18	‘I didn't spray much myself but I was aware of farmers locally using sprays.’

### 3.1 Occupation‐Related Pesticide Exposure and PD Risk

A significant feature of many participants' experiences with the use of or exposure to pesticides was that this was related to their occupation, which, for many, was farming or plant/veterinary science. This theme captures individuals' lived experiences of exposure to neurotoxic pesticides such as paraquat, rotenone and organophosphates. Individuals reported on the occupational vulnerability of agricultural and industrial environments, where pesticides are commonplace. The findings underscore the need for stronger regulatory policies, worker education and alternative pest management strategies to mitigate PD risk in high‐exposure professions. People throughout the interviews held beliefs about the onset of PD based on their local environment:

‘she is 73 now so she was diagnosed maybe about 13 years ago and she lives in a small cul de sac in (town name redacted), and 2 other people were diagnosed with Parkinson's three of them often wondered was it anything to do with the local fertilizer factory in (town name redacted), which is since closed but would have released a lot of chemicals back in the day’

#### 3.1.1 Type of Pesticide Used

While not all interviewees provided exact pesticide names, using terms like ‘chemicals’, ‘pesticides’ and ‘sprayers’ as umbrella terms, some provided specific examples related to their occupations. Interviewees frequently mentioned specific pesticides such as paraquat, rotenone and organophosphates, describing their routine use in farming, pest control and industrial applications.

One interviewee, who worked in the discipline of Botany and Plant Science, recalled being in contact with ‘*quite a few chemicals’*. These ‘chemicals’ were recounted as being:

‘Dichlorodiphenyltrichloroethane [DDT], Copper sulphate, Glyphosate and many more herbicides, pesticides and a whole range of veterinary pharmaceuticals.’

Another participant, a veterinarian with PD, described the regular use of a combination of insecticides and fungicides to create treatments in the form of baths for animals. Consequently, the bathing of animals resulted in exposure of this individual to a plethora of pesticides:

‘Organophosphates (foxim, diazinon, tetrachlorvinphos), Organochlorines (DDT), Amitraz, Piretroids and Enilconazole.’

Another study participant described their experiences of working with a commonly used herbicide formulation, ‘Forefront’, which contains aminopyralid and triclopyr:

‘…Forefront. It comes in a 1ltr can. I was spraying it at about 7:30 one evening and there was some dew out that night. I was in the field the next morning, and I saw that the weed had gone down. It was at 6 o'clock the following morning, so it can work that quick, in the space of 8‐10 hours. It is the most expensive grass spray that you can buy. It is very very effective, and it is commonly used…’

In some cases, it was evident that participants had used chemicals for which they had little or no knowledge of the chemical agents or their potential toxic effects. Many participants expressed limited awareness of the neurotoxic effects of these chemicals, often recounting unprotected exposure due to inadequate safety training or lack of personal protective equipment:

‘A few times we did use “bluestone” sometimes. We mixed powders, I think they were blue and white powders, in a big tin bath with a stick and then pour it into a watering can before pouring it onto weeds.’

#### 3.1.2 Duration and Frequency of Exposure

The use of pesticides was a regular feature in the everyday working lives of many participants. The experiences of those PwP who were farmers indicate that exposure to pesticides was frequent, since they described prolonged exposure to pesticides over years or even decades, often beginning in early adulthood and continuing without significant protective measures. Many workers reported daily or seasonal pesticide application, with repeated handling and inhalation. Some participants recalled working in environments where pesticide residues lingered in the air or soil or on crops, leading to chronic low‐dose exposure. Others mentioned increased exposure during peak agricultural seasons, when pesticide use was more frequent and intense:

‘I was a cereal grower all my working life and would have been a user of agrichemicals and pesticides for all those years.’

Pesticide use is described as a necessary and frequent feature of both tillage and sheep farming. Interviewees described frequent and prolonged exposure to pesticides used in sheep farming, particularly organophosphate‐based insecticides and dips for parasite control. Many farmers and farmworkers recounted handling these chemicals regularly over several decades, often without adequate protective equipment or awareness of long‐term health risks. Some reported intensive exposure during peak treatment periods, such as sheep‐dipping seasons, when they repeatedly immersed animals in pesticide‐laden solutions.

‘My father … was a sheep and tillage farmer for most of his life … harsh chemicals used for sheep foot‐bathing…’

A veterinarian recounted the frequency of their exposure during 15 years of veterinary practice. They described direct skin contact, inhalation of chemical fumes and accidental splashes while treating animals, particularly during sheep‐dipping procedures and pesticide application. These exposures were without adequate protective measures, over long working hours, with inadequate ventilation and limited awareness of long‐term neurological risks:

‘I was exposed 2‐3 times per week *to insecticides and fungicides*’

Many of these PwP participants spent the larger part of their lives being regularly exposed to a variety of pesticides, raising concerns about the cumulative effects of chronic low‐dose pesticide exposure:

‘…been in contact with quite a few chemicals used in weed and pest control for more than 50 years.’

### 3.2 Dangers of Pesticides and Barriers to PPE Usage

The use of PPE was mentioned in some of the statements from those participants who used pesticides in relation to their occupation, as discussed above. While most mentions of PPE were by those whose occupation involved pesticide contact, some farmers with PD who were interviewed did not mention any use of PPE. They reported inconsistent or inadequate protection due to discomfort, lack of availability or time constraints. Many participants working in agriculture, sheep farming and veterinary practice recalled handling pesticides with minimal protective gear, often relying on basic gloves or masks that offered insufficient protection against inhalation and skin absorption. It was notable that the descriptions by those whose occupations involved pesticide exposure did not reflect any regulations, strict guidelines or mandatory use of suitable equipment during pesticide exposure. For example:

‘At the time I was only using medical gloves, though I frequently ended up bathed myself—once you are surrounded by sheep shaking themselves, you get the bath too.’

‘During my 47 years of farming I have used different pesticides, often without mask or gloves, using a pump action garden sprayer.’

In some of the interviews, the lack of PPE was discussed in relation to the use of pesticides by the interviewee's family member, who had been diagnosed with PD. Some interviewees expressed limited awareness of the importance of PPE, while others highlighted employer negligence, citing a lack of proper training or enforcement of safety protocols:

‘He did have a toxic type exposure to a pesticide last year—he was spraying without PPE.’

Participants were aware of the dangers of pesticides, and implemented PPE in their work and life:

‘I think there is an increase in people using it yes. I have a spray cabinet at home, with all of the PPE in it. People are more aware now than they were 10 years ago, of the dangers’

Another individual PwP described some level of improvement in the extent of PPE use between generations within a family:

‘A cloth (like a head scarf) or a scarf was used when my father was spraying. When I took over later, I took a bit more precautions, like a proper mask and gloves. I worked as a stone mason for a number of years and as such came in contact with cement dust a lot, I can remember breathing it in when opening bags. On the farm, lime was used commonly as a disinfectant. It was scattered in cattle houses after they had been cleaned. You would throw it around and the only thing to protect you from the dust was your collar pulled up over your nose and mouth. I was diagnosed with PD in 2013, it was early onset idiopathic PD.’

Similar statements emerged from several participants, indicating a lack of awareness about the importance of PPE use in previous generations of their families. This highlights stricter safety regulations, increased access to high‐quality PPE, and enhanced training programs to protect workers from the long‐term neurotoxic effects of pesticide exposure:

‘Back in the day (1960's/70's) people didn't take much notice of “health and safety”. I can remember farmers mixing chemicals in tin baths with their hands, no gloves. When they would pull their hands out, they would be brown up to where it went in. It was actually a crime, years ago, to have ragwort (*Jacobaea vulgaris*) on your land, you could be fined by the Guards. People picked them, again no gloves, and put them in a pile before burning them. I can remember a lot of burning; weeds, rubbish, rubber, gorse. There was little health and safety back then. I was unaware of the link.’

‘I grew up on a farm, I would not have been involved in the spraying much but I do remember it happening from time to time. Back then there was no real PPE like there is now, it was a scarf or a tea towel wrapped around your head covering your nose and mouth.’

‘We … put socks or bags tied with twine over our arms and went pulling weeds/ragwort.’

There was a general theme indicating that awareness and use of PPE have improved over time and that the current generation of agricultural workers is generally more informed about PPE and uses it more than their parents did:

‘…Me and my husband didn't really understand the dangers of these chemicals, but we were careful. When my son took over in the 80's/90's, he was very careful with chemicals and sprays.’

While most mentions of PPE were by those who were in contact with pesticides due to their occupation, some participants were exposed to pesticides incidentally. These individuals with PD were exposed to pesticides in their daily lives outside of their occupation or against their will and therefore were not using PPE.

In one case, the lack of PPE usage when using pesticides was due to misinformation and misconceptions about the dangers of pesticide exposure, leading to direct, long‐term exposure to a product containing rotenone:

‘My Dad gave me a puffer canister of ‘ABOL derris dust’ to control woodlice and ants…. I remember being concerned after inadvertently breathing in some of the dust. However, Dad assured me that I had no need to worry as it was a natural product which was safe to use. His view was not surprising because ABOL had been traditionally marketed as a “non‐poisonous insecticide”. Being confident of its safety, I continued to use it occasionally for about 20 years.’

Participants exposed to pesticides, presumably without PPE, due to family or community factors will be discussed further in the next section.

### 3.3 Family and Community Influence/Mixed Messaging About Pesticide Safety

As discussed above, some participants were subject to second‐hand exposure to pesticides over the long term, mainly due to their countryside home location or their parents' occupation. For example, some PwP participating in this study were children of farmers and were exposed to pesticides in the air when they were sprayed on the farm. Many interviewees reflected on the way in which family traditions and community norms around pesticide use in farming or agricultural work influenced their own behaviours and their perceptions of risk:

‘The farm I grew up on had a tillage operation that relied heavily on pesticides at the time.’

‘I am … from a farming background.’

Several participants described how their exposure was unprecedented, unavoidable and therefore unprotected due to their countryside location or lifestyle:

‘I live in a village surrounded by agricultural fields…. I smell pesticides in the air when they apply it—glyphosate, insecticide and fungicides—and several times I've been out cycling and have encountered a sprayer at close quarters.’

Similarly, other participants discussed countryside exposure to pesticide usage in the locality. For example, one who had developed PD at an early age said:

‘I also live in the countryside where pesticides would be used.’

‘Yeah so (my mother) is 73 now so she was diagnosed maybe about 13 years ago and she lives in a small cul de sac in (town name redacted), and 2 other people were diagnosed with Parkinson's, the three of them often wondered was it anything to do with the local fertilizer factory in (town name redacted) which is since closed but would have released a lot of chemicals back in the day’.

Another PwP described long‐term childhood exposure to a variety of pesticides due to their father's role for 30 years as the Chief Technical Officer of a large chemical company:

‘Dad always had a company car for his work, which doubled as the family car. He used the car to transport chemical concentrates, dressed grain, and work equipment including a knapsack spray pack and protective gloves and goggles. He would often offload these and store them in the garage, sometimes for prolonged periods.’

This participant with PD described close‐proximity exposure to the transport and storage of the pesticides involved in his father's work:

‘…I recall containers of Reglone and Gramoxone being stored on the upper shelves in the garage.’

This childhood exposure to pesticides also extended to regular use of these agents as gardening products in the family garden:

‘Dad also acquired a large range of garden product samples from (name of the company in which he worked redacted), including Pathclear, Roseclear, blue Slug Pellets (metaldehyde) and Derris Dust (rotenone). Dad … would regularly treat the roses against aphids and fungi and treat the paths and lawn against weeds.’

It was interesting that some participants described several PwP within their family:

‘Yes, there is (history of Parkinson's in family). My grandad had Parkinson's, he was diagnosed in his late 50's early 60's, and he was a thatcher by trade, and he had a small holding, like a farm. He unfortunately had to stop thatching because of it. He is from a fairly big family and I think 2 of his siblings, and my father's 1st cousins had Parkinson's. There is also a strong history of Dementia in my family as well, Lewy body dementia. One of his brothers had Parkinson's and Dementia. That was all on my father's side. On my maternal side there is no Parkinson's but on the paternal side there is’

Some participants also noted that they received mixed messaging from medical consultants or health care professionals when they had queried a potential link between pesticides and PD:

‘He did have a toxic type exposure to a pesticide last year—he was spraying without PPE and was very dizzy and weak afterwards, his chronic symptoms seemed to start after that exposure (dizziness, rigidity and tremor). We said this to our Consultant but he said there is no link.’

## 4 Discussion

This analysis explored the experiences of individuals with PD regarding their pesticide exposure, and identified three primary themes: ‘Occupation‐Related Pesticide Exposure and PD Risk’, ‘Dangers of Pesticides, and Barriers to PPE Usage’, and ‘Family and Community Influence’. These themes reflect several issues in relation to both the frequency and nature of pesticide exposure in Ireland. This study seek to address a critical gap in the existing literature, as there is a lack of qualitative studies that examine how individuals who are exposed to pesticides construct their understanding of PD and relate it to environmental risks. While the state of the art is comprehensive in linking pesticide use to various health issues, particularly neurodegenerative diseases such as Parkinson's, these studies predominantly rely on quantitative methods that measure the prevalence and correlation between exposure and disease onset. Few studies have explored the lived experiences of individuals and the social construction of their disease in the context of occupational pesticide exposure. By using qualitative methodologies such as interviews and thematic analysis, this study aimed to explore how workers, such as farmers, perceive and interpret the relationship between their environment and their PD diagnosis. Understanding how people navigate and make sense of their exposure to pesticides within their specific workplace contexts is crucial for gaining deeper insights into the subjective experience of PD. Additionally, this study explores how family, community and social norms influence these perceptions, providing a more holistic view of the complex interplay between environmental risks and health outcomes. This qualitative approach fills a significant gap in current research by highlighting the personal narratives and psychosocial dimensions of pesticide‐related health risks, which are often overlooked in epidemiological studies.

### 4.1 Occupation‐Related Pesticide Exposure and PD Risk

This qualitative data highlighted a potential role for occupational exposure to pesticides in the development of PD, from the perspectives of farming families living with a PD diagnosis. Many participants recounted frequent and extensive contact with various pesticides due to their professional activities, which in this cohort was mostly farming. This frequent exposure came with varying, but generally low, levels of understanding about the types of pesticides to which individuals were exposed, and of the associated risks. While some participants could specify the types of pesticides used, others referred to them in more general terms. This lack of ability to precisely identify the pesticides being used strongly indicates a need for education regarding types of pesticides and their uses, as well as the potential risks involved with each. Being able to recognise and categorise the types of pesticides that are in common usage could be vital for avoiding harmful exposure to pesticides, particularly co‐exposure to multiple agents, as it is known that certain combinations of pesticides can exacerbate the risk of PD [19]. Furthermore, a lack of information regarding pesticide usage in occupational settings poses a challenge for further research on links between specific chemicals and PD, or indeed other diseases. Accurate information acquired from users is vital to such research.

Overall, the experiences of the participants in this study corroborate existing literature, which indicates that prolonged exposure to pesticides accelerates both the onset and progression of PD [2, 26]. Many qualitative studies have focused on agricultural workers, particularly farmers and pesticide applicators, revealing that long‐term exposure to pesticides contributes to a heightened risk of developing PD [23, 27]. Interviews with workers often reveal a normalisation of pesticide use within the agricultural industry, where individuals perceive pesticide application as an integral and unavoidable part of their work [27, 28]. Future avenues should focus on education and outreach to these farming communities and on gathering more accurate data about the types and combinations of pesticides being used. Identifying which pesticides, or combinations of agents, contribute most significantly to PD risk will be crucial to inform targeted interventions for those working in agricultural and botanical occupational fields.

### 4.2 Dangers of Pesticides and Barriers to PPE Usage

Another identified theme was the inadequate use of PPE. Participants' PPE usage was either unmentioned, minimal or non‐existent. For instance, some individuals reported using only basic medical gloves or no protective gear at all, leading to direct and frequent contact with the chemicals being used. This insufficient protection is concerning given the well‐established link between pesticide exposure and many adverse health conditions, including PD [17, 29, 30]. The variability in PPE usage among participants points towards a broader issue of a lack of awareness and adherence to safety guidelines [30]. Additionally, qualitative research points to a lack of consistent PPE use and inadequate training, which exacerbate the health risks associated with pesticide exposure [27, 31, 32, 33]. Appropriate PPE is essential for reducing exposure risks; this may vary from thin dermal coverings such as gloves, boots, long‐sleeved tops, and coveralls, depending on the type of pesticides used and on the preferences of individual users. However, our interview data highlighted that current practices in this cohort fall short of those recommended [29]. In the future, improved education about PPE usage and enforcement of stricter regulations will be important to ensure that appropriate protective measures become standard practice when working with pesticides.

### 4.3 Family and Community Influence/Mixed Messaging About Pesticide Safety

This third theme was identified because several broader environmental and social factors that contributed to pesticide exposure emerged from the analysis of the interviews. Many participants described growing up in farming environments or rural areas where pesticide use was prevalent. This exposure was often unregulated and unavoidable, affecting both adults and children. For example, one participant described childhood exposure due to their father's work with pesticides, which included handling and storing chemicals within family spaces. Social and community influences also shape workers' perceptions of pesticide use and health risks, with many participants reporting that family traditions and community norms around pesticide application prevented them from recognising the full extent of the hazards they face [20, 28, 31]. A significant gap in public health protocols is evident, as pesticide exposure is not adequately addressed in terms of warnings or restrictions. The widespread nature of exposure demands a more comprehensive public health strategy to manage and mitigate the impact of pesticides in both occupational and residential contexts.

In the future, the handling of pesticides should be coupled with education on the associated risks, guidelines and responsibilities that come with their use. The correct use of PPE should be mandatory whenever pesticides are used. Those applying pesticides must be mindful of others who may be affected by their use and should minimise the harmful effects wherever possible. This precaution will help to ensure that others are not unknowingly or unwillingly subjected to the effects of pesticides.

Moreover, with a view to reducing future pesticide usage, the adoption of farming practices that minimise the need for chemical interventions could be a solution. By implementing ecological methods, such as crop rotation, promoting natural pest predators and enhancing biodiversity in agricultural spaces, farmers can significantly decrease their reliance on pesticides [29, 34]. This transition from synthetic to agroecological practices could aid in protecting those who use pesticides in occupational settings, as well as those in the surrounding communities, from the harmful effects of exposure, fostering a healthier environment overall. Our research explored how family, community and social norms influence these perceptions, providing a more holistic view of the complex interplay between environmental risks and health outcomes. This qualitative approach fills a significant gap in current research by highlighting the personal narratives and psychosocial dimensions of pesticide‐related health risks, which are often overlooked in epidemiological studies.

## 5 Limitations

These interviews were conducted using a semi‐structured approach, gathering each participant's own lived experience and perspectives on the topic of the potential relationship between PD and pesticide use, which resulted in varying responses. Structured demographic data were not collected due to the nature of the interview process. The study may be subject to selection bias due to participants' selection from biased information sources, such as rural settings. The study primarily relies on qualitative data and thus may not be representative of the broader population of workers exposed to pesticides. There may also be a possibility of a recollection bias by participants of any exposure in a given condition. Participants were asked to reflect on their past pesticide exposure and health experiences, which may have been influenced by their current health status or memory distortions and may have led to inaccurate or selective reporting.

## 6 Conclusions

This study reveals significant occupational and environmental exposure to pesticides among PwP from farming backgrounds in Ireland, highlighting inadequate use of PPE and unregulated exposure to pesticides. This echoes findings from studies in other countries and adds to the existing evidence for a significant role of exposure to environmental toxicants in the rapid global increase in PD incidence [12]. These findings emphasise an urgent need for enhanced education on pesticide risks, as well as stricter safety protocols and regulatory measures. To mitigate risks, it is essential to provide knowledge about pesticides and their associated dangers. Mandatory use of appropriate PPE should be enforced whenever pesticides are handled, protecting both users and those who might be exposed inadvertently. Additionally, adopting farming practices that reduce reliance on chemical intervention, such as crop rotation, promoting natural pest predators and enhancing biodiversity, have potential to significantly decrease pesticide use and the associated harm to individuals and communities. Addressing these issues could help reduce the incidence of PD linked to pesticide exposure. Future initiatives should focus on identifying specific pesticides used and the exposure levels involved, and on developing targeted interventions to minimise risk within agricultural communities.

## Author Contributions

**Lucy M. Collins:** conceptualisation, methodology, investigation, formal analysis, writing – original draft, data curation. **Sarah Moore:** writing – original draft, data curation, formal analysis. **Emma O'Shea:** writing – review and editing, methodology. **Aidan White:** software, data curation. **Aideen M. Sullivan:** writing – original draft, supervision, resources, project administration, conceptualisation, methodology, investigation, funding acquisition.

## Ethics Statement

Ethical approval for this study was obtained from the University College Cork Social Research Ethics Committee (Log no. 2021‐116). Confidentiality and anonymity were maintained, and data were securely stored to protect the participants' identities. All interviews were conducted with trust and respect between researchers and participants.

## Consent

Informed consent was obtained from all individuals, ensuring that each participant understood the purpose of the study, how their data would be used and their right to withdraw at any time.

## Conflicts of Interest

The authors declare no conflicts of interest.

## Data Availability

The data that support the findings of this study are available on request from the corresponding author. The data are not publicly available due to privacy or ethical restrictions.
